# Notified dengue deaths in Myanmar (2017-18): profile and diagnosis delays

**DOI:** 10.12688/f1000research.23699.1

**Published:** 2020-06-09

**Authors:** Nwe Ni Linn, Khine Wut Yee Kyaw, Hemant Deepak Shewade, Aye Mon Mon Kyaw, Myat Min Tun, San Kyawt Khine, Nay Yi Yi Linn, Aung Thi, Zaw Lin

**Affiliations:** 1 Vector Borne Disease Control Programme, Department of Public Health, Ministry of Health and Sports, Nay Pyi Taw, Myanmar; 2 Department of Operational Research, International Union Against Tuberculosis and Lung Disease (The Union), Mandalay, Myanmar; 3 Center for Operational Research, International Union Against Tuberculosis and Lung Disease (The Union), Paris, France; 4 Department of Operational Research, The Union South East Asia, New Delhi, India

**Keywords:** Dengue fever, Mortality, Delay in diagnosis, Severe dengue, Operational Research, SORT IT

## Abstract

**Background: **Complications in dengue usually occur between day four and day six after fever onset. Hence, early diagnosis and haematological monitoring are vital. Among all hospital reported dengue deaths in Myanmar in 2017-18, we assessed the i) patient profile, ii) proportion of patients who arrived with a dengue diagnosis at admission and iii) delays in diagnosis after fever onset.

**Methods: **This was a descriptive study involving secondary data. For all the notified deaths, death investigation forms were not available in prescribed format and therefore, data were extracted from hospital case records.

** Results:** Of 304 deaths, 184 (60.5%) were female and 233 (76.6%) were less than 10 years old. Township level hospitals or below reported 36 deaths (11.8%) and the remaining deaths were from higher level facilities. Dengue was diagnosed before admission in 26 (8.5%) people and 169 (55.6%) were in shock at admission. Of 208 with date of fever onset recorded, the median diagnosis delay was four (interquartile range-IQR: 3, 5) days. Patient level delay (median three days) was a major contributor to the diagnosis delay.

**Conclusions:** Most of the patients who died did not have a diagnosis of dengue before admission. This calls for an urgent review of health system preparedness in peripheral health facilities to suspect, diagnose, monitor, refer and treat dengue in children and patient level factors for better understanding of the reasons of delay. Timely filling of death investigation forms in a prescribed format and quarterly death reviews based on these is recommended.

## Introduction

Dengue is a mosquito-borne viral disease that has rapidly spread in tropical and subtropical regions. Around 3.9 billion people are at risk of dengue in 128 countries where there is good evidence of dengue occurrence ^1^. In recent years, transmission has increased predominantly in urban and semi-urban areas and the incidence of dengue in adults is increasing ^2, 3^. In 2016, there were a total of 3.3 million reported dengue patients ^4^.

Severe dengue usually occurs between day four and six after fever onset (called the critical stage, during which fever subsides) and is one of the leading causes of hospitalization and death among children and adults in most Asian and Latin American countries ^4^. There are four distinct serotypes of dengue virus (DEN-1, DEN-2, DEN-3 and DEN-4). People who recover from dengue infection may get lifelong immunity against one particular serotype and cross-immunity for a few months. Subsequent infections by other serotypes increase the risk of developing severe dengue ^4^. Half a million people with severe dengue require hospitalization every year, and around 2.5% of them die ^4^. Mortality is highest in younger age groups and reduces with increasing age ^5^.

Dengue mortality can be reduced by early detection and good referral systems especially at the primary health care level, predicting and managing severe dengue with appropriate treatment at the hospital level, reorienting health services to cope with dengue outbreaks, and training health personnel at all levels of the health system ^6^. Once the diagnosis is confirmed or suspected, severe dengue can be detected early by clinical (significant abdominal pain, persistent vomiting, lethargy, restlessness, mucosal bleeding, fluid accumulation) and haematological monitoring ^7, 8^.

Delay in diagnosis could be at the level of the patient or health system ^9, 10^. Late presentation is associated with severe disease in adult dengue patients ^10^. Dengue death is commonly associated with co-morbidities and clinicians should be aware if dengue patients fulfil the severe case definition on admission ^11, 12^.

Myanmar is a high dengue burden country in the Asia Pacific Region. Between 2011 and 2015, of the 89,832 dengue related admissions, 97% were children ^13^. There is limited published literature on diagnosis delays after fever onset among children or adults who died due to dengue. Therefore, we aimed to describe the profile and delays in diagnosis among all dengue deaths in Myanmar.

## Methods

### Study design and population

We conducted a descriptive study involving secondary data collection. We included all dengue deaths reported to the Vector Borne Disease Control (VBDC) Programme in Myanmar during 2017–18.

### Setting

Myanmar, a tropical country, is located in the Southeast Asia Region, bordering the Republic of China on the north and northeast, Laos on the east, Thailand on the southeast, Bangladesh on the west, and India on the northwest. Myanmar is still a predominantly rural country, with around 30% of the population living in urban areas ^14^. The country is divided administratively into Nay Pyi Taw union territory and 14 states and regions and consists of 74 districts and 330 townships ^15^.

Doctors are first available at sub-township level station hospitals. Basic health staff at the level of rural health centres (below station hospital) and sub-centres (below rural health centre) provide comprehensive primary health care. Specialists are available at district level hospitals.

### Diagnosis and management of dengue

SD BIOLINE dengue rapid test kits (includes NS1 Antigen, IgG antibody and IgM antibody) and packed cell volume (PCV) using a centrifuge machine (microhaematocrit or haematocrit) are available at some of the station hospitals. PCV and platelet counts using haematology auto analysers are available at district hospitals and above. The NS1 antigen ELISA test is available only at National Health Laboratory, Yangon, and is used for evaluation of rapid test kits. Diagnosis, treatment and monitoring of dengue patients in health care facilities highly depends on the availability of doctors (at station hospitals), rapid test kits, centrifuge machine and complete haemogram (for platelet count). The laboratory facilities may not be available as assigned or sometimes may malfunction. All the severe or complicated dengue (confirmed or suspected) patients are referred to the nearby district, regional or tertiary hospital.

In 2011, the Myanmar Paediatric Society published Paediatric Management Guidelines (second edition) and updated them in 2018 ^16^. The chapters for dengue were adopted from the 2009, 2011 and 2012 WHO guidelines ^8, 12, 17^. In 2018, the National Guideline for Clinical Management of Dengue for doctors and basic health staff were published ^18, 19^. Training has been provided to basic health staff and doctors since 2018 in selected states and regions based on funding availability. It is being expanded to the whole country from 2019.

### Surveillance of dengue

The assistant director or team leader is responsible for VBDC Programme planning and implementation at the state and regional levels. All hospitals maintain a dengue register for admitted patients. The VBDC works closely with the department of medical services to collect data on dengue morbidity and mortality by using dengue register. In case of a laboratory confirmed dengue with any grading at hospital, VBDC staff, basic health staff and community volunteers carry out dengue prevention and control activities within a 100 meter radius of the patient’s house.

If there is any dengue death, hospitals are expected to inform VBDC staff immediately. VBDC staff go to the hospital and fill out a dengue death investigation form and report back to the central VBDC office.

Dengue patients that do not get admitted are not reported to the VBDC. The dengue patients managed by the private sector are not routinely reported to the programme, except one private hospital which reported one dengue death during 2017–18.

### Variables and data collection

A line list of all dengue deaths was prepared in March 2019 by using the dengue death reports submitted to central VBDC Programme by State and Regional offices. Though we were planning to extract data from death investigation forms, it was found that they were not filled out in the prescribed format. Therefore, data were extracted from the hospital case records to a structured data collection form. These paper-based hospital case records were sent to principal investigator.

The data collected included age, sex, state or region, dengue grading at admission, hospital name, date of admission, type of diagnosis (laboratory/clinical), type of first health care provider visited and cause of death. The dates of onset of fever, first health care provider visited after fever, diagnosis, admission and death were also collected. Operational definitions for ecological regions and dengue grading have been summarized in Table 1.

**Table 1.** Operational definitions used in this study ^3, 13, 18^.

Variable		*Definition*
Ecological regions	Delta and low land	*Heavy rainfall more than 2500 mm. Includes Ayeyarwady, Yangon, and Bago regions; Mon* *and Kayin states*
	Hills	*Moderate to heavy rainfall. Includes Kachin, Kayah, Chin, and Shan states*
	Coastal	*Heavy rainfall more than 2500 mm. Includes Rakhine state and Taninthayi regions*
	Plains	*Uneven topography and rainfall less than* *1000 mm. Includes Magway, Mandalay, Sagaing, and Nay Pyi Taw regions*
Dengue Grading	Dengue Fever	*Fever with two of the following*• *Headache.* • *Retro-orbital pain.* • *Myalgia.* • *Arthralgia/bone pain.* • *Rash.* • Haemorrhagic manifestations Hess* test + > 70% • *No evidence of plasma leakage*
	DHF Grade I	*Fever and haemorrhagic manifestation* *Hess test + > 90%* *Evidence of plasma leakage*
	DHF Grade II	*As in Grade I plus spontaneous bleeding.*
	DHF Grade III	*As in Grade I or II plus circulatory failure* *(weak pulse, narrow pulse pressure (≤20 mmHg), hypotension, restlessness).*
	DSS (Hypotensive shock)	*As in Grade III plus profound shock* *with undetectable blood pressure and pulse*
	Expanded Dengue Syndrome	*Complications of severe profound shock or associated with underlying host conditions/* *diseases or coinfections. Central nervous system manifestations including convulsions,* *spasticity, changes in consciousness and transient paresis have been observed.*

DHF, dengue hemorrhagic fever; DSS, dengue shock syndrome.

### Data entry and analysis

The data were double-entered and validated using EpiData entry software (version 3.1, EpiData association, Odense, Denmark) and analysed using STATA (version 14.2 College Station, Texas). The profile was summarized using frequencies and proportions. Patient level (fever onset to first health care provider visit), health system level (first health care provider visit to diagnosis) and total diagnosis delay (fever onset to diagnosis) were calculated. The hospital stay duration was calculated from date of admission to date of death. The duration and delays in days were summarized using the median and interquartile range (IQR).

### Ethical statement

Administrative approval for the study was obtained from the VBDC Programme and ethics approval was received from Ethical Review Committee, Department of Medical Research, Myanmar (Ethics/DMR/2018/149 dated 7 December 2018) and Ethics Advisory Group, The Union, Paris, France (EAG number 45/18 dated 23 August 2018). As this study involved review of routinely collected secondary data, a waiver for informed consent was sought and approved by the ethics committees. This study involved the use of patient medical data in which all data analysed were anonymized.

## Results

Of 31,288 and 23,273 dengue patients hospitalized in 2017 and 2018, 192 (0.6%) and 112 (0.5%) died, respectively.

### Patient profile of dengue deaths

The characteristics of these 304 reported deaths at admission and cause of death are described in Table 2. Of 304 deaths, 184 (60.5%) were among females. The median age was six (IQR: 3, 9) years, 233 (76.6%) deaths were among children (<10 years) and 246 (80.9%) were reported during the wet (June to October) season. Dengue shock syndrome at admission was seen in 169 (55.6%) and at death in 257 (84.5%). Of 304 deaths, 36 (11.8%) were reported from the township level hospital or below and the remaining were from higher level facilities.

**Table 2.** Profile of dengue deaths reported to the national programme in Myanmar (2017–18).

Characteristics		N	(%)
Total		304	(100.0)
Age group (years)			
	0–4	110	(36.2)
	5–9	123	(40.5)
	10–14	37	(12.2)
	15–30	24	(7.9)
	31–45	5	(1.6)
	<45	5	(1.6)
Sex			
	Male	120	(39.5)
	Female	184	(60.5)
Severity at admission			
	DHF grade I	60	(19.8)
	DHF grade II	18	(5.9)
	DHF grade III	47	(15.5)
	DSS	169	(55.6)
	EDS	5	(1.6)
	Not recorded	5	(1.6)
Type of hospital where death was reported ^¥^			
	Station hospital	3	(1.0)
	Township hospital	33	(10.9)
	District hospital	85	(28.0)
	Regional hospital	78	(25.7)
	Teaching hospital	19	(6.3)
	Tertiary hospital	85	(28.0)
	Private hospital	1	(0.3)
Ecological region			
	Delta and lowland	165	(54.3)
	Hills	29	(9.5)
	Coastal	40	(13.2)
	Plains	70	(23.0)
Seasonal			
	Hot (March to May)	39	(12.8)
	Wet (June to October)	246	(80.9)
	Cool (November to February)	19	(6.3)
Type of first health care provider visited			
	Direct admission to hospital	57	(18.8)
	Government doctor	9	(3.0)
	Private doctor	26	(8.5)
	BHS and paramedical staff	7	(2.3)
	Pharmacy, untrained person, self-medication	32	(10.5)
	Station or township hospital	31	(10.2)
	Places not specified	142	(46.7)
Cause of death			
	DSS	257	(84.5)
	EDS	40	(13.3)
	Dengue encephalitis	5	(1.6)
	Others	1	(0.3)
	Not recorded	1	(0.3)

DHF, dengue hemorrhagic fever; DSS, dengue shock syndrome; EDS, expanded dengue shock syndrome; BHS, basic health staff.

^¥^Station hospital = hospital covering villages in a township; township hospital = hospital covering whole township; district hospital = hospital covering the whole district; regional hospital = hospital covering the whole state/region; teaching hospital = hospital that provides medical education and training to health professionals; tertiary hospital = hospital providing tertiary care, which is health care from different specialists.

The type of first health provider visited after fever onset was not recorded for 142 (46.7%) patients and 57 (18.8%) got directly admitted to hospital where they died. Among patients who had a first health care provider visit (that was recorded) before getting admitted to the hospital where they died (n=105), 32 (30.5%) either went to a chemist or untrained provider or self-medicated ( Table 2).

### Diagnosis delay after fever onset

The date of onset of fever was only available for 208 (68.4%) patients. Of these 208, the patients were admitted after a median of four (IQR: 3, 5) days after fever onset. The median patient level delay was three (IQR: 1, 4) days and median health system level diagnosis delay was zero (IQR: 0, 2) days. Total diagnosis delay was four (IQR: 3, 5) days ( Table 3).

**Table 3.** Diagnosis delays and admission time among dengue deaths reported to the national programme in Myanmar (2017–18).

Delay	Times taken (days) between	N *	Median	(IQR)
Patient level diagnosis delay	Onset of fever and first healthcare provider visit	130	3	(1,4)
Health system level diagnosis delay	First healthcare provider visit and diagnosis	149	0	(0,2)
Total diagnosis delay	Onset of fever and diagnosis	206	4	(3,5)
Time to admission	Onset of fever and admission	208	4	(3,5)
Admission time	Admission and death (hospital stay)	304	1	(0,2)

IQR, interquartile range. *Includes those for whom dates were available.

**Figure 1.**
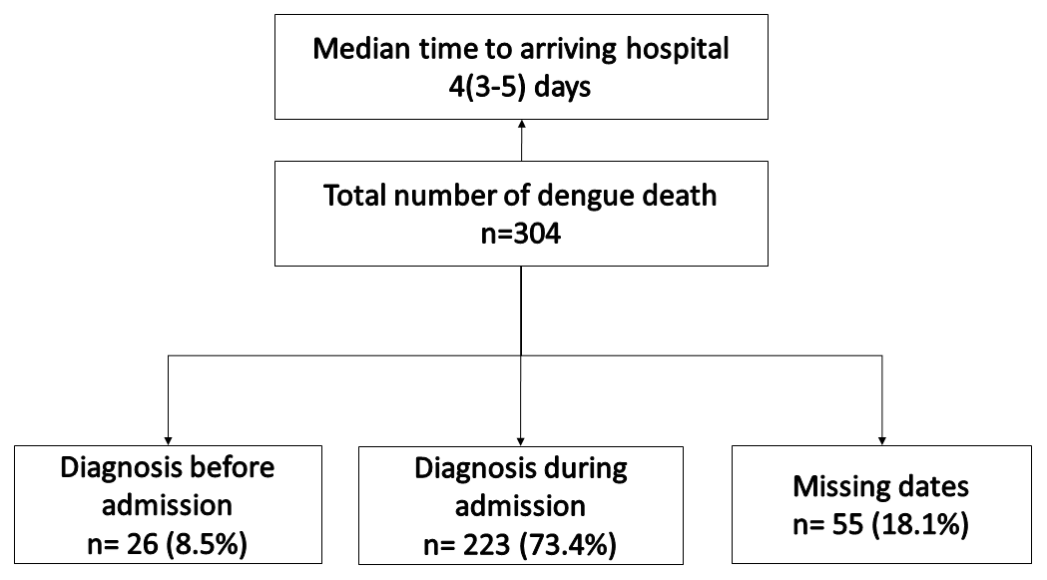
Timing of dengue diagnosis in relation to hospital admission among dengue deaths reported to the national programme in Myanmar (2017–18).

Of 304, 81 (26.6%) patients died within 24 hours of admission. Before admission, dengue fever was diagnosed in only 26 (8.5%) ( Figure 1).

## Discussion

This is the first published study from Myanmar that described diagnosis delay from fever onset among all reported dengue deaths in Myanmar.

Most of the patients did not have a diagnosis of dengue fever before admission and more than half of the patients were in shock at admission. This was also seen in a study from Malaysia ^20^. In Brazil, among patients with severe dengue, having a laboratory diagnosis was associated with a lower chance of dying when compared to not having a diagnosis ^21^.

Absence of or delay in dengue fever diagnosis or lack of suspicion of dengue before admission may not have given time for proper treatment. The patients may have self-referred or were referred to higher level facilities when clinical features worsened, resulting in patients directly presenting with severe dengue at hospitals. Early diagnosis could have ensured detection of patients who were more prone to develop dengue haemorrhagic fever and dengue shock syndrome. Complications or worsening of dengue usually occurs at or after four days of fever onset ^8, 23^. This is when the fever also subsides.

Patient level delay (median three days) was a major contributor to the total diagnosis delay. Studies from Myanmar have reported that most people with fever sought care from untrained personnel or self-medicated and the reasons for avoiding care from trained health care providers (basic health staff, village health volunteers) was that minimal action was taken if fever patients were not diagnosed with malaria ^24, 25^.

The training for doctors and basic health staff on updated dengue guidelines was done only in 2018 and all regions were not covered. This could have contributed to the lack of clinical suspicion of dengue in peripheral health facilities.

The highest numbers of patients were reported in the wet season and were from the less than 10 years old age group. This highly correlates with the epidemiology of dengue disease ^2, 5^. The rainy season is also the school start time in Myanmar.

### Implications for policy and practice

This study has five implications for policy and practice. First, a review of health system preparedness to suspect, diagnose, monitor, refer and treat dengue is required at peripheral health facilities as most patients were admitted without a diagnosis of dengue fever. This includes review of availability of rapid test kits at station hospitals and above. Considering the burden of dengue is similar to malaria in Myanmar, availability of test kits should be ensured at the level of station hospitals and preferably up to the grass roots level for evaluation of undifferentiated fever ^17^. Training of basic health staff and doctors of all regions regarding the updated 2018 national guidelines is required. Guidelines for the management of undifferentiated fever should be developed ^26^. This will especially be beneficial for health care providers in peripheral health facilities.

Second, as death investigation forms were not filled out in the prescribed format for all dengue deaths, we recommend quarterly dengue death reviews at regional and national levels. This will indirectly ensure that states and regions fill out the dengue investigation forms in the prescribed format and submit them to the VBDC Programme regularly.

Third, we recommend a similar study among all the reported dengue patients to confirm whether these findings are present (or not). Fourth, to address patient level delay there is a need to raise awareness in the community regarding health seeking for undifferentiated fever. Finally, the following four indicators may be added to the monthly forms in the health management information system in health facilities: i) total suspected with dengue ii) total confirmed dengue and iii) diagnosis within three days of fever among confirmed dengue.

### Strengths and limitations

We collected nationwide data of all reported dengue deaths and this represents the ground reality. Data were robust as they were double entered and validated.

There were two major limitations in the study. First, death investigation forms were not being filled out. The reason for this is not known. This resulted in many missing variables as these were not available in the hospital case records. This includes details on the first health care provider visited and various dates. Diagnosis delay related findings are limited by the absence of the date of fever onset in one-third of patients. These forms also help the programme at a national level to review the causes of death, identify preventable health system related causes and take timely action.

Second, the variables recorded in hospital represent the clinical condition of the patients and other important variables for dengue prevention and control such as previous episodes of dengue, comorbidities, housing, income, occupation/school places, travel history, utilization of bed net, use of mosquito repellent were not routinely available.

To conclude, this study reported a long diagnosis delay after fever onset among dengue deaths reported over two years in Myanmar. This calls for an urgent review of health system preparedness to suspect, diagnose, monitor, refer and treat dengue at peripheral health centres and patient level factors for better understanding of reasons for diagnosis delay. Filling out of dengue death investigation forms should be ensured and corrective action be taken through regular regional and national dengue death reviews.

## Data availability

### Underlying data

Figshare: Nwe Ni Linn et al 2020 dataset v2. https://doi.org/10.6084/m9.figshare.12355958.v1 ^27^

This project contains the following underlying data:

- Data_NNL.xlsx (all de-identified variables extracted for this study, alongside a codebook explaining all fields and field values).

Data are available under the terms of the Creative Commons Zero "No rights reserved" data waiver (CC0 1.0 Public domain dedication).
