# Supplementary material for: Notified dengue deaths in Myanmar (2017-18): profile and diagnosis delays
Source: F1000Res. 2020 Jun 9;9:579. [Version 1] doi: 10.12688/f1000research.23699.1 (PMC7336047; doi:10.12688/f1000research.23699.1)
Supplement: Peer review history

# Peer review: Reviewer response for version 1

**DOI:** 10.5256/f1000research.26148.r64470

**Published:** 2020-07-15

**Authors:** Sangay Zangmo, Lila Maya

**Affiliations:** 1 Royal Centre for Disease Control, Ministry of Health, Thimphu, Bhutan

This manuscript projects interesting findings and correlations on diagnosis delay with deaths of dengue patients in Myanmar. For a country with high dengue burden, interventions on such findings will greatly enhance dengue diagnosis, referral, timely treatment and patient survival. Methods are clearly elucidated; strengths and weaknesses have been described well.

 Language and command in general is good, however, may need to be re-looked into in some sentences. I have some suggestions as below:

1. In the abstract: *Most of the patients who died did not have a diagnosis of dengue before admission. *This Sentence needs rephrasing *.*
2. In the introduction:
*Dengue death is commonly associated with co-morbidities and clinicians should be aware if dengue patients fulfil the severe case definition on admission*. Word “severe” may be in inverted commas
3. In the methods:
*Doctors are first available at sub-township level station hospitals.*
  Remove “first”

Some general comments:

1. Definition of “delay” is not clear.  After how many days would it be considered as delayed? In table 3, would it be more appropriate to use the word “duration” since there is no clear definition as to from which day “delay” would imply?
2. Ecological factors in this manuscript described include rainfall. Rainfall can be one of the factors among other such as temperature, vegetation and urbanization.  Information on these variables, if available may be interesting. Some papers have been cited for your reference.
3. In the discussion: *Mo*st *of the patients did not have a diagnosis of dengue fever before admission and more than half of the patients were in shock at admission.*
  Does that mean patient previously visited some kind of health centres prior to admission?
  For those that were diagnosed, how was the diagnosis made?

Is the work clearly and accurately presented and does it cite the current literature?

Partly

If applicable, is the statistical analysis and its interpretation appropriate?

Yes

Are all the source data underlying the results available to ensure full reproducibility?

Yes

Is the study design appropriate and is the work technically sound?

Yes

Are the conclusions drawn adequately supported by the results?

Yes

Are sufficient details of methods and analysis provided to allow replication by others?

Yes

Reviewer Expertise:

Molecularand virological techniques, Molecular epidemiology

We confirm that we have read this submission and believe that we have an appropriate level of expertise to confirm that it is of an acceptable scientific standard, however we have significant reservations, as outlined above.

## References

[1] : Spatial and temporal patterns of dengue incidence in Bhutan: a Bayesian analysis. *Emerg Microbes Infect*.2020;9(1) : 10.1080/22221751.2020.1775497 1360-1371 10.1080/22221751.2020.1775497 32538299PMC7473275

# Peer review: Reviewer response for version 1

**DOI:** 10.5256/f1000research.26148.r65627

**Published:** 2020-07-03

**Authors:** Zhuo Lin Chong

**Affiliations:** 1 Institute for Public Health, National Institutes of Health, Ministry of Health, Kuala Lumpur, Malaysia

**Title and abstract:** Reflective of the content

**Introduction:** The study is important. It would be perfect if the flow of the introduction can be rearranged to make the justification of the study stands out. Beside that, there were many references to adults and children in the text, with emphasis on the latter. Suggest to realign them to better reflect the title and content of the article.

**Objectives: **They are clearer in the abstract but in not the main text. Suggest to detail them out in the main text as well. 

**Methods: **

1. Setting: The second paragraph on the availability of healthcare personnel is important in the interpretation of this study. Suggest making it clearer to readers not familiar with Myanmar healthcare system. E.g. replace the below hospital and center in bracket with an additional sentence right after the mention of sub-township level station hospitals to make clear the organisation. 
2. Variables and data collection: Would be great if more details are given on the data collector/extractor, any quality control in place, how missing values were handled.  

**Results: **

1. Suggest to state if the number of dengue death extracted for this study matched the number of death reported to VBDC through surveillance. Response rate is important to assess selection bias. 
2. Table 2: Type of first health care provider visited has categories that overlap each other, e.g. directly admitted into hospital and government doctor can be the same thing. Please label the categories more distinctively. Would be perfect if they are also defined in the operational definition. 
3. Patient profile of dengue deaths: Suggest: i) to remove "Among patients who had a first health care provider visit (that was recorded) before getting admitted to the hospital where they died (n=105)", ii) use 32 (10.5%), and iii) use "pharmacist" instead of chemist. Discrepancies can create confusion and reduce readability.
4. Figure 1 Timing of dengue diagnosis: If the objective for this comes in second, suggest to present it in the same way. Consider additional subsection corresponding to the objective. Also, number and percentage of patients who died within 24 hours of admission could not be found in this figure.
5. Diagnosis delay after fever onset: Suggest to convert this table into a figure featuring a horizontal bar, on the left end of which labelled fever onset, the right end - death, and each stage in between. Then put your N, median, and IQR below with tracking lines going upward to different stages on the bar. That way it is understandable at a glance.

**Discussion:**

1. 2nd paragraph: "Most of the patients did not have a diagnosis of dengue fever before admission and more than half of the patients were in shock at admission. This was also seen in a study from Malaysia." Please make it clear that the reference to Malaysia is pointing to "shock at admission" and not "did not have a diagnosis of dengue fever before admission". As 73.6% of patients were suspected to have dengue in the Malaysian study.
2. The first section of discussion can be strengthened by comparing the results of this study with that of the other studies in depth. 
3. Implication for policy and practice: Strengthening the discussion in the first section would make these recommendations more persuasive. Suggest to add good practices from other countries to make it compelling. 

**References:** To reorganize references. Combine identical item into one reference, e.g. WHO Global Strategy 2012 in references 6 & 8.

Is the work clearly and accurately presented and does it cite the current literature?

Partly

If applicable, is the statistical analysis and its interpretation appropriate?

Yes

Are all the source data underlying the results available to ensure full reproducibility?

Yes

Is the study design appropriate and is the work technically sound?

Yes

Are the conclusions drawn adequately supported by the results?

Yes

Are sufficient details of methods and analysis provided to allow replication by others?

Yes

Reviewer Expertise:

Evaluation of dengue rapid diagnostic test kits, dengue surveillance

I confirm that I have read this submission and believe that I have an appropriate level of expertise to confirm that it is of an acceptable scientific standard.

# Peer review: Reviewer response for version 1

**DOI:** 10.5256/f1000research.26148.r64469

**Published:** 2020-06-18

**Authors:** Jian-Wei Xu, Hui Liu

**Affiliations:** 1 Yunnan Institute of Parasitic Diseases, Puer, China; 2 Yunnan Institute of Parasitic Diseases, Pu'er City, China

Dengue is now ranking as one of the most critical global mosquito-borne viral diseases and is endemic in over 100 countries. For many countries, dengue is becoming a threat to their public health, and further adversely impacting their health services and economies.  Early diagnosis and effective supporting treatment for dengue  can reduce transmission and improve patient prognosis. Data on death, delayed treatment are rare. This paper gives reader a profile and diagnosis delays in Myanmar. It is value to get indexed.

 However, we suggest the author consider:

1. The part of introduction is not strong enough, not make the objectives clear.
2. "Most of the patients who died did not have a diagnosis of dengue before admission", please make clear in method section, "how were these patients confirmed as dengue ? just based on records of  symptoms or have laboratory test evidence?
3. More discussing in detail on reasons of delayed diagnosis and potential solution, this will be significance on dengue control in Myanmar.

Is the work clearly and accurately presented and does it cite the current literature?

Partly

If applicable, is the statistical analysis and its interpretation appropriate?

Yes

Are all the source data underlying the results available to ensure full reproducibility?

Yes

Is the study design appropriate and is the work technically sound?

Partly

Are the conclusions drawn adequately supported by the results?

Yes

Are sufficient details of methods and analysis provided to allow replication by others?

Yes

Reviewer Expertise:

Control of vector borne diseases

We confirm that we have read this submission and believe that we have an appropriate level of expertise to confirm that it is of an acceptable scientific standard.

## References

[1] : Dengue viruses circulating in Indonesia: A systematic review and phylogenetic analysis of data from five decades. *Rev Med Virol*.29(4) : 10.1002/rmv.2037 e2037 10.1002/rmv.2037 31099110
